# Bladder cancer with urinary diversion by a sigmoid colon conduit after transverse colon stoma

**DOI:** 10.1002/iju5.12665

**Published:** 2023-11-08

**Authors:** Tadashi Aoki, Ryota Furuya, Masanari Fukasawa, Munehiro Nozawa, Yoshio Takihana, Makoto Sudoh, Hiroshi Nakagomi

**Affiliations:** ^1^ Department of Urology Yamanashi Kosei Hospital Yamanashi Japan; ^2^ Department of General Surgery Yamanashi Kosei Hospital Yamanashi Japan

**Keywords:** chemotherapy, indocyanine green (ICG), quality of life (QOL), sigmoid colon conduit, transverse colon stoma

## Abstract

**Introduction:**

Sigmoid conduit is one of the methods for achieving urinary diversion, but it is performed less frequently than ileal conduit and ureterostomy. Herein, we report a case in which a sigmoid colon conduit was performed after nephrostomy and transverse colostomy.

**Case presentation:**

A 70‐year‐old man was referred to our hospital because of a bladder tumor. Computed tomography and transurethral biopsy revealed advanced bladder cancer with ureteral and rectal invasion. Despite drug therapy, the tumor progressed. Thus, nephrostomy and transverse colostomy were performed for urinary and fecal diversion, respectively. Subsequently, chemotherapy was administered for 8 months. As nephrostomy‐related complications occurred frequently during chemotherapy, a sigmoid colon conduit was performed instead of nephrostomy for urinary diversion to improve the patient's quality of life.

**Conclusion:**

In patients with advanced bladder cancer requiring a double stoma of the urinary and fecal tracts, sigmoid colon conduit may be selected as a urinary diversion method.

**Abbreviations & Acronyms**
CT: computed tomography
ICG: indocyanine green
QOL: quality of life

**Keynote message**

With the advancements in drug therapy for bladder cancer, a good long‐term prognosis may be achieved. Therefore, some cases will require a double stoma of the urinary and fecal tracts. This may result in an increase in cases of not only ileal but also colonic conduits for urinary diversion.

## Introduction

One of the methods for urinary diversion is a sigmoid colon conduit.^1^ However, its indications are limited, such as after a total pelvic exenteration. Additionally, it is performed less frequently than an ileal conduit^2^ or a ureterostomy. We performed nephrostomy as a urinary diversion method for ureteral obstruction and a transverse colostomy as a fecal diversion method for rectal obstruction in a patient with bladder cancer. Herein, we report a case in which a sigmoid colon conduit was performed to improve the patient's QOL.

## Case presentation

**Fig. 1:**
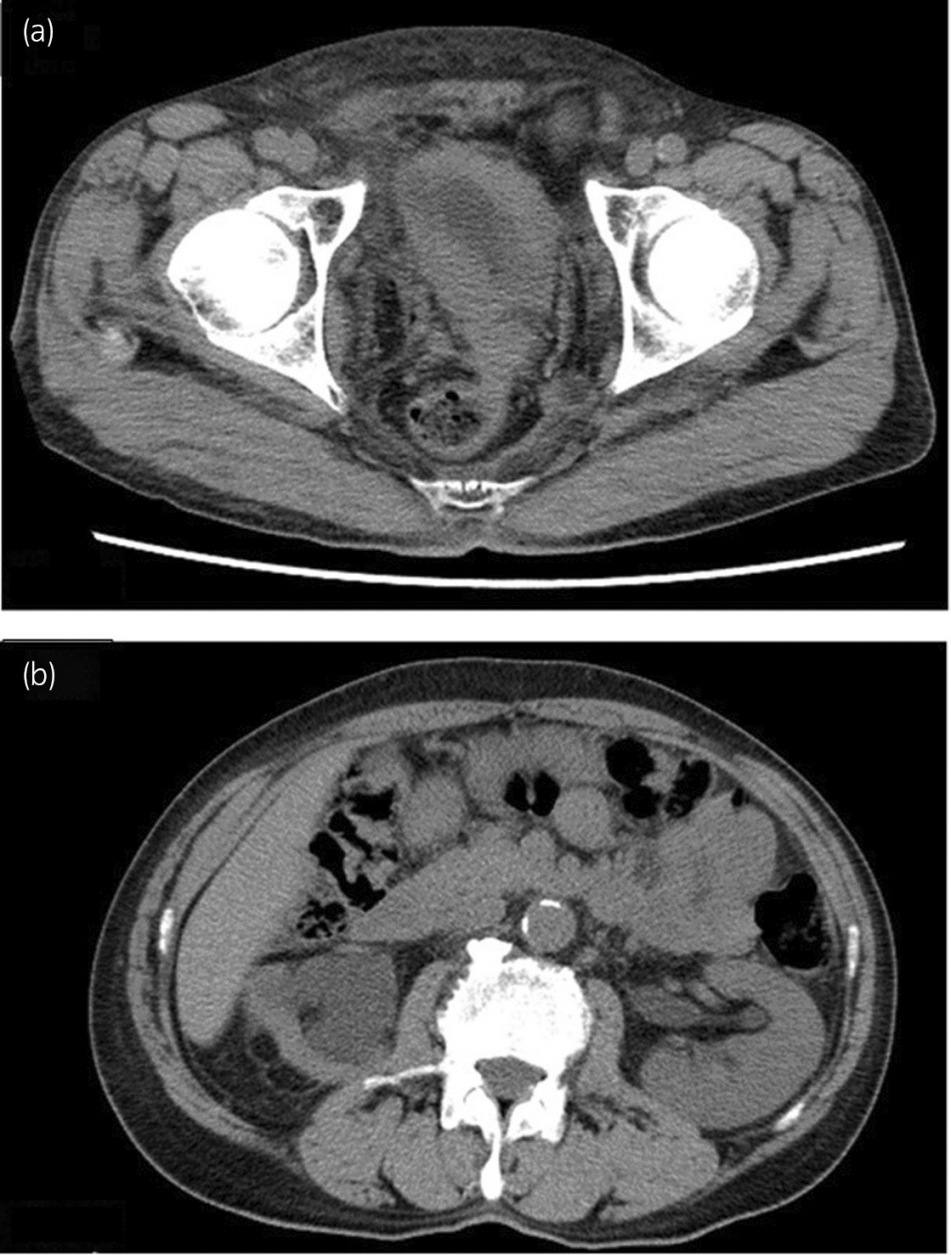
CT shows a thickened bladder wall and contiguous tumor extending into the rectum (a) as well as bilateral hydronephrosis and right renal atrophy (b).

A 70‐year‐old man presented to his primary care physician with the chief complaint of gross hematuria. A bladder tumor was identified via ultrasonography, and he was referred to our hospital. CT indicated circumferential thickening of the bladder wall and a soft mass shadow contiguous with the bladder extending to the rectum and pelvic wall (Fig. 1a). Moreover, hydronephrosis and renal atrophy on the right side and hydronephrosis on the left side were noted (Fig. 1b).

**Fig. 2:**
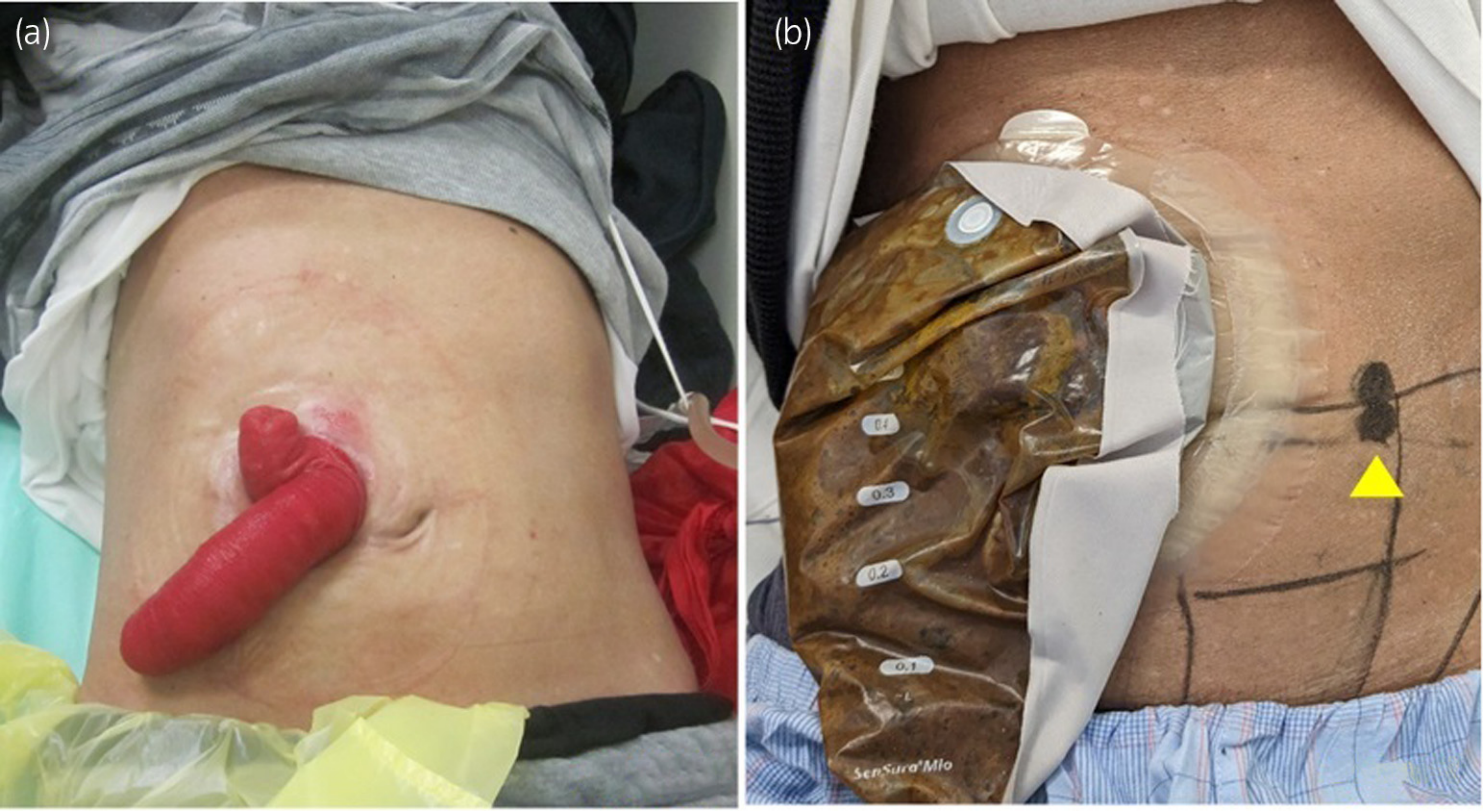
(a) A transverse colonic stoma was constructed in the right upper abdomen, in which prolapse of the stoma developed. (b) A urinary stoma was planned for the left abdomen (arrowhead).

Transurethral bladder tumor biopsy confirmed a high‐grade urothelial carcinoma with stratified squamous metaplasia, but distant metastases were not observed. The patient refused local treatments, such as surgery and radiotherapy. The patient was in a state of renal failure and underwent dialysis, but there was hope that his renal function would recover. Therefore, gemcitabine and paclitaxel were administered as first‐line chemotherapy because these drugs have lower nephrotoxicity than carboplatin, which has a higher nephrotoxic potential.^3^, ^4^ After one cycle, his renal function recovered and the patient no longer required dialysis. At the end of four cycles, the bladder cancer shrank and the left hydronephrosis resolved. However, approximately 4 months later, the tumor progressed and the left hydronephrosis recurred, which led to post‐renal renal failure. Thus, left nephrostomy was performed. Subsequently, five courses of secondary pharmacotherapy with pembrolizumab were administered. However, the tumor continued to progress and caused rectal obstruction; thus, a rectal stent was placed. Thereafter, third‐line chemotherapy with enfortumab vedotin was initiated. After three courses, the rectal wall invaded by the tumor collapsed, which led to abscess formation between the rectum and the bladder; thus, a transverse colonic stoma was constructed. Seven more courses were administered, and the tumor reduced in size at 11 months. However, nephrostomy‐related complications occurred frequently during therapy. Three episodes of acute pyelonephritis necessitated hospitalization. To improve the patient's QOL, a sigmoid colon conduit was performed as a urinary diversion method instead of nephrostomy. Owing to the presence of a prolapsed transverse colonic stoma in the right upper abdomen (Fig. 2a), the plan was to construct a urinary stoma in the left abdomen (Fig. 2b).

**Fig. 3:**
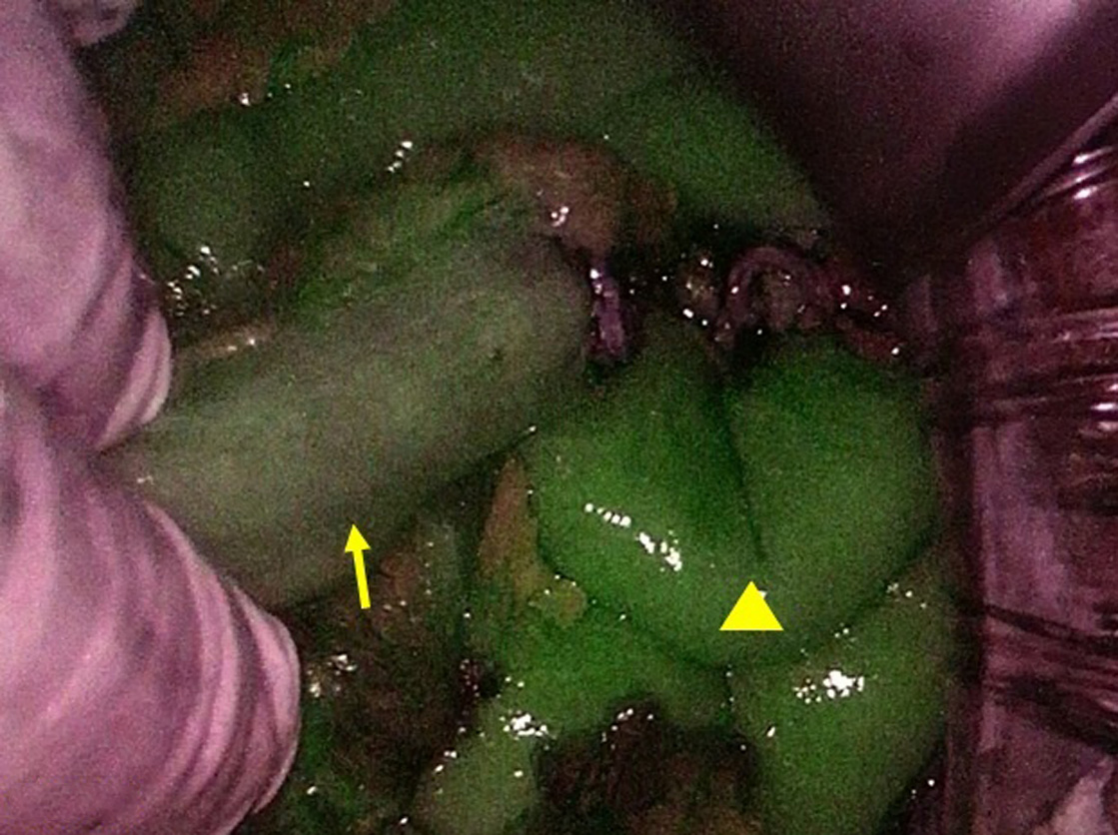
ICG fluorescence method was used to evaluate the blood flow. Compared with the ileum (arrowhead), the free sigmoid colon (arrow) had less blood flow; however, it was sufficient as a conduit.

**Fig. 4:**
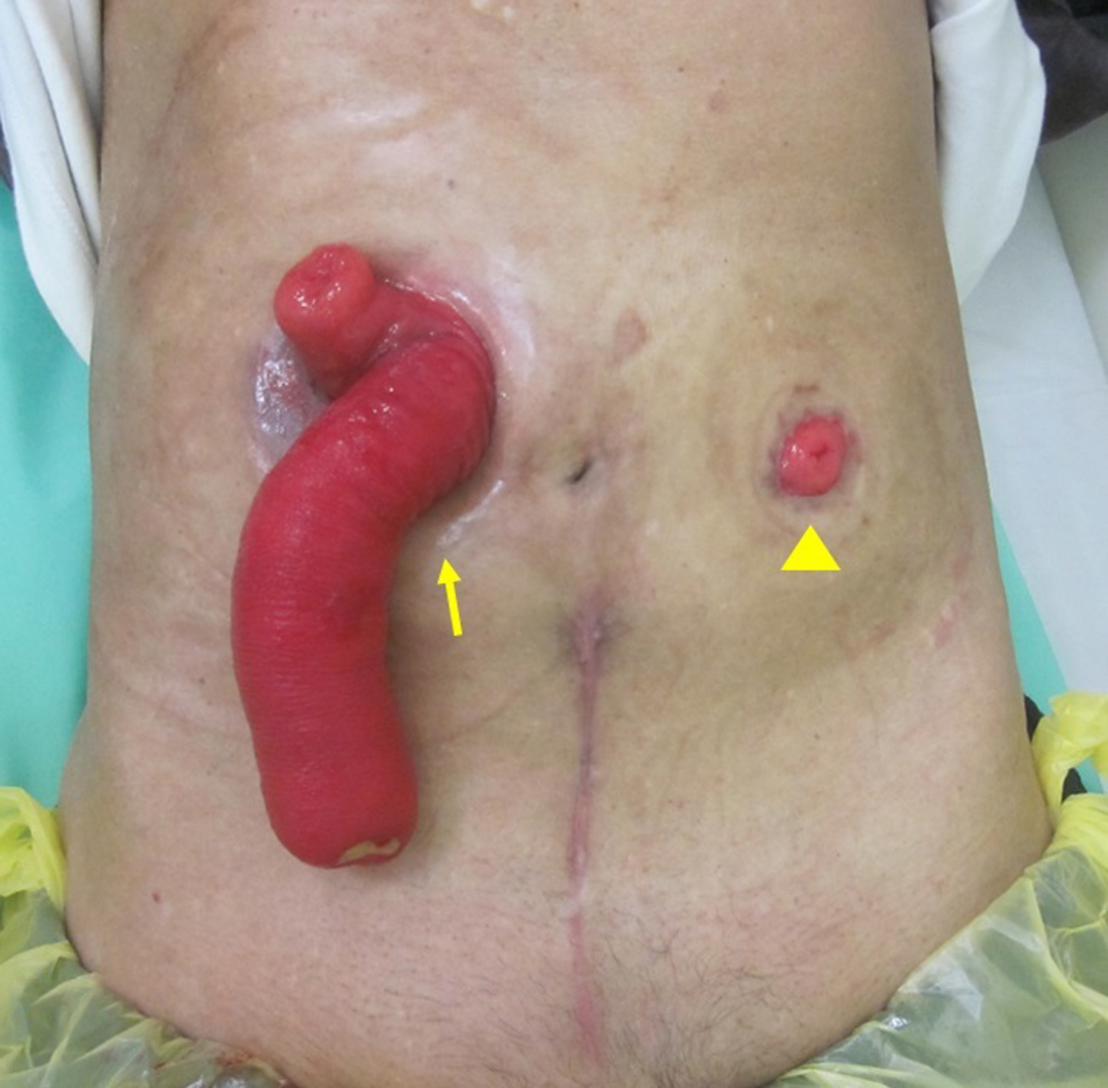
A transverse colonic stoma (arrow) in the right upper abdomen; a sigmoid colon stoma (arrowhead) was constructed in the left abdomen.

Under general anesthesia, a midline lower abdominal incision was made. The sigmoid colon was identified and proximally traced to the transition with the descending colon. The left ureter was dorsolaterally identified in that area. The maximum separation of the normal ureter was approximately 6 cm as the retroperitoneal space was covered with inflamed tissue. Next, the sigmoid colon was separated to identify the site for stoma creation. Longer freed sigmoid colon, for example, 20 cm, was needed, but only 10 cm could be isolated because of strong inflammatory adhesion in the lower pelvis. To evaluate the blood flow in the isolated sigmoid colon, ICG fluorescence method was used (Fig. 3). ICG (2.5 mg/mL) was administered intravenously 5 mL. The freed ureter and sigmoid colon were both shorter than expected. Therefore, a submucosal tunnel was not formed; however, a ureteral conduit anastomosis was created and a ureteral stent was placed. As planned, a stoma was created on the left abdomen (Fig. 4). At this time, the nephrostomy was not removed.

Postoperative complications included intestinal obstruction, ureteral leakage, and wound infection, which resolved spontaneously. Both the ureteral stent and nephrostomy were removed, and the patient was discharged 30 days after the surgery. Outpatient chemotherapy was resumed and continued.

## Discussion

In recent years, new therapeutic agents have emerged and the long‐term prognosis for patients with advanced‐stage bladder cancer is more promising than before.^5^, ^6^ This situation requires urinary management to enhance patients' QOL. An ileal conduit is commonly performed as a urinary diversion using the intestinal tract; however, in this case, we constructed a sigmoid colon conduit. One of the advantages of this method is that as the colon wall is thick, ureteral anastomosis with submucosal tunneling^7^ can prevent ductoureteral reflux. Additionally, it is anatomically easy to create a stoma in the sigmoid colon, whether on the left or right side. However, one disadvantage is that the sigmoid colon has poor blood flow and mobility compared with the ileum, which is considered a risk factor for stoma necrosis and intestinal anastomotic failure.

In this case, the sigmoid colon was the first choice owing to the following reasons: (i) it was not used from the descending colon after the transverse colon was constructed, (ii) the length of the ureter that can be separated was expected to be limited, (iii) the stoma had to be constructed in the left abdomen, and (iv) the patient was under chemotherapy and wished to resume it early after the surgery.

The ileal conduit was not considered as the first choice because a new intestinal anastomosis was required, and when creating a stoma on the left side of the abdomen, the position of the free ileum and ureteral conduit anastomosis should be considered.^8^ Although ureterostomy is minimally invasive, it can shorten the free ureter and is difficult to perform.

ICG fluorescence was useful to evaluate the blood flow in the sigmoid colon conduit. The method has been employed to reduce anastomotic leak in colon cancer surgery.^9^

Intestinal obstruction, ureteral leakage, and wound infection were among the postoperative complications. These are not unique to the sigmoid colonic conduit and could occur with a relatively high frequency in the ileal conduit too.^10^

We could not conclude whether an ileal conduit with an intestinal anastomosis or a sigmoid conduit without an intestinal anastomosis has a better safety profile. However, in this case, no major complications were noted, urinary diversion was successfully performed, the patient was freed from the nephrostomy, and a good QOL was maintained. No pyelonephritis or renal function deterioration was observed postoperatively.

## Conclusion

More and more patients with advanced‐stage bladder cancer are expected to have a good long‐term prognosis with the advent of novel drug therapies. Cases, such as the one reported one, requiring a double stoma of the urinary and fecal tracts are likely. In such instances, the sigmoid colon conduit may be selected as a urinary diversion method.

## Author contributions

Tadashi Aoki: Conceptualization; investigation; visualization; writing – original draft. Ryota Furuya: Writing – review and editing. Masanari Fukasawa: Writing – review and editing. Munehiro Nozawa: Writing – review and editing. Yoshio Takihana: Writing – review and editing. Makoto Sudoh: Investigation; writing – review and editing. Hiroshi Nakagomi: Investigation; project administration; supervision; writing – original draft.

## Conflict of interest

The authors declare no conflict of interest.

## Approval of the research protocol by an Institutional Reviewer Board

Not applicable.

## Informed consent

The patient provided informed consent for publication of this case report.

## Registry and the Registration No. of the study/trial

Not applicable.
